# Senior volunteers: addressing loneliness in times of COVID-19

**DOI:** 10.1007/s10433-023-00788-5

**Published:** 2023-10-20

**Authors:** Zaira Torres, Sara Martínez-Gregorio, Amparo Oliver

**Affiliations:** Department of Methodology of the Behavioral Sciences, Faculty of Psychology, University of Valencia, Av. de Blasco Ibáñez, 21, 46010 Valencia, Spain

**Keywords:** Loneliness, Volunteer, COVID-19, SHARE

## Abstract

Volunteering in old age plays an important role in addressing feelings of loneliness, but little research has been conducted on the impact of engaging in volunteering activities during the COVID-19 pandemic. This study investigates the longitudinal impact of volunteering on feelings of loneliness. We analyzed data from 31,667 adults aged 50 years and older in the Survey of Health, Ageing and Retirement in Europe (SHARE), across three consecutive waves (one before the pandemic and two during the COVID-19 pandemic). Binary logistic regression analyses were conducted for loneliness, using volunteering and several control variables as independent variables, and found that even after controlling for previous loneliness, volunteering has a protective effect against experiencing feelings of loneliness. Those who participated in volunteering activities before or during the second pandemic period had a lower risk of loneliness during the second pandemic period. Volunteering during the second pandemic period, before and during the second pandemic period, and during all three periods measured in the study was negatively associated with the odds of feeling lonely. Encouraging volunteering among older adults can be a useful strategy to prevent loneliness during future emergency situations like the COVID-19 outbreak.

**Supplementary Information:**

The online version contains supplementary material available at 10.1007/s10433-023-00788-5.

## Introduction

Even before the COVID-19 pandemic, scholars and public health officials had already been warning of a potential "loneliness pandemic", with loneliness among older adults identified as a significant public health concern (Gerst-Emerson and Jayawardhana 2015). Loneliness refers to the multifaceted set of feelings, cognitions and adverse emotions that arise when a person's social needs are not being met in terms of quantity or quality of social relationships (Campagne 2019; Ernst and Cacioppo 1999; Hawkley and Cacioppo 2010). It is important to note that loneliness, the perceived subjective experience of social isolation (Campagne 2019; Hawkley et al. 2009) is different from objective social isolation, which is usually defined as a measure of network size or the objective absence of interactions with family and friends (Courtin and Knapp 2017; Victor et al. 2000). Thus, in our study, we refer to loneliness as this evaluation of the match between the quantity and quality of existing relationships and one’s relationship desires (Cacioppo and Hawkley 2009; De Jong-Gierveld and Havens 2004).

Lonely is no trivial matter, it is considered a public health problem that can have adverse effects on both the physical and mental health of adults (Coyle and Dugan 2012). Loneliness has been associated with an increased risk of cardiovascular disease (Valtorta et al. 2018), depression (Domènech-Abella et al. 2021), cognitive decline (Boss et al. 2015), frailty (Kojima et al. 2022), and even premature mortality (Kristensen et al. 2022). The COVID-19 outbreak led many countries to implement stay-at-home policies and physical distancing measures to contain the spread of the disease. The focus of these measures was to protect older people, who, if infected, are more vulnerable to a severe disease progression or even death (Posch et al. 2020). However, these measures seem to have worsened pre-existing feelings of loneliness among adults (Arpino et al. 2022; Atzendorf and Gruber 2022; Bu et al. 2020; Krendl and Perry 2021).

Participating in volunteering activities in old age can be an effective way to alleviate feelings of loneliness (Carr et al. 2018; Cho and Xiang 2022; Kim et al. 2020; Lee 2022a; Sundström et al. 2021). Volunteering involves engaging in unpaid, non-compulsory activities, either through an organization or by directly benefiting others (Taniguchi 2012). Based on the integrated theory of volunteer work of Wilson and Musick (1997), volunteer work is conceptualized as a collective behavior as well as a productive and ethically guided work that requires human, social, and cultural capital. Volunteering is also defined as various forms of assistance or helping behaviors that individuals offer willingly (Callow 2004). However, volunteering is not only a behavior motivated by altruism. It can be motivated by a variety of reasons (Snyder et al. 2000). For example, some people aim to express prosocial and humanitarian values through action; others seek to learn more about the world, people, and their own abilities; some volunteer to feel fulfilled; others seek to distract themselves from their own problems; others want to strengthen ties with others; and finally, some seek paid employment opportunities (Stukas et al. 2016).

Older people may feel lonely due to a variety of factors that can be addressed by participating in volunteer activities. For example, loneliness may occur due to unmet standards of social relationships and social activity (Tesch-Romer and Huxhold 2019). Engaging in social activities is considered to help reduce these feelings of loneliness, as it promotes a sense of belonging (Niedzwiedz et al. 2016; Sirven et al. 2015). Also, older people are particularly vulnerable to feelings of loneliness due to a lack of meaningful role and status in family and society. Older adults may experience a loss of social roles throughout their lives due to retirement, changes in family structure (e.g., becoming a widowed) or experience physical limitations (Singh and Misra 2009). Therefore, engaging in volunteer work is considered to provide an opportunity for developing a new social role thus sharing of skills and life experiences (Chambre 1984; Papa et al. 2019) and promoting a sense of reciprocation and purpose of life (Lee 2023).

Research has suggested that volunteering during the COVID-19 could improve some psychosocial out- comes. For example, Sin et al. (2021) used convenient sampling from Canada and the USA and found that older people who engaged in informal support had lower levels of anger and frustration during the COVID-19 pandemic. In another study, Chan et al. (2021) examined a sample of older people from China and discovered that one specific volunteering activity, assisting others in purchasing daily necessities, was associated with fewer depressive and anxiety symptoms. However, these studies are cross-sectional, used convenient sampling. Moreover, although anger, anxiety and depression are related to loneliness, they are not the same concept. So more specific investigation addressing loneliness is need.

To our knowledge, only one study specifically investigates the effect of volunteering on feelings of loneliness in older people during the COVID-19 pandemic. This study found that the disruption of volunteering since COVID-19 has had a negative impact on volunteers. Also, they found that the individuals who stopped volunteering were more likely to report feeling lonely compared to those who still volunteering (Biddle and Grey 2021). Even though this study is a great contribution to the literature, presents some limitations. The study of Biddle and Grey (2021) is focused only on a sample of people from Australia, so the results of this study cannot be generalized to other countries. Also, even the study has a longitudinal design, it does not include pre-COVID data on loneliness, so they only can achieve if those who stopped volunteering were far more likely to say that they felt lonely at least some of the time than those who remained as volunteers. Lastly, this study only examined the early stages of the pandemic (April 2020- April 2021). Therefore, the main objective of our study is to explore the potential protective benefits of volunteering on feelings of loneliness before, during, and after the initial months of the pandemic in engagement on feelings of loneliness in a representative sample of adults aged 50 years and older from Europe and Israel, across three consecutive waves of the SHARE project (one before the pandemic and two during the COVID-19 pandemic).

Volunteering in old age may help individuals alleviate loneliness. Nevertheless, due to new social distancing regulations implemented by governments during the pandemic, but traditional forms of volunteering have also become more challenging, especially for older adults (Lachance 2021). As a result, volunteer opportunities have decreased significantly, and some studies have reported that the participation of older people in volunteering activities has declined considerably (Biddle and Grey 2021; Chatzi et al. 2020; Grotz et al. 2020; Principi et al. 2022). Other studies, however, have found that older people were more likely to volunteer in times of COVID-19, despite the risk to their health and distancing measures (Chan et al. 2021; Mak and Fancourt 2022). Also, before the COVID-19, some studies showed that the intensity and continuity in the volunteer activities was related to lower loneliness (Carr et al. 2018; Cho and Xiang 2022). So, considering that fact, we aim to analyze whether people in our sample volunteer during COVID-19 and whether the action of participating in volunteer activities is related to feelings of loneliness.

In addition, when studying the relationship between volunteering and loneliness, it could be important to consider the characteristics of the volunteers. For example, the study by Carr et al. (2018) showed that volunteering may be a particularly important for alleviating loneliness among older adults who were recently widowed. Research on characteristics of volunteers showed that usually there are higher rates of volunteering among older adults with higher education and economic status, better physical and mental health, and lower age (Butrica et al. 2009; Curvers et al. 2018; Dury et al. 2015). However, in the context of the COVID-19 pandemic, the desire to provide support during the crisis may have encourage older people who would not previously volunteer. Therefore, to better understand the relationship between volunteer activity and the loneliness feelings, we aim to investigate the characteristics of the volunteers in three time points.

## Method

### Participants

We use data from the Survey of Health, Ageing and Retirement in Europe (SHARE) (SHARE; Börsch-Supan et al. 2013), a longitudinal survey of individuals aged 50 and above conducted every 2 years since 2004, covering 27 European countries and Israel. SHARE data recollection was approved by the Ethics Council of the Max Planck Society and all participants provided informed consent. This study combined data from the 8^th^ wave of the SHARE survey, the COVID Survey 1, and COVID Survey 2.

The 8^th^ wave (Börsh-Supan 2022a), part of the regular SHARE data, was collected between October 2019 and March 2020 using a computer-assisted personal interviewing (CAPI) method. The COVID waves were special modules administered to assess the impact of the pandemic, COVID Survey 1 (Börsch-Supan 2022b) was conducted between June and September 2020 and COVID Survey 2 between June and August 2021 (Börsch-Supan 2022c). Both collected data through computer-assisted telephone interviewing (CATI).

Our analytic sample comprised 31,667 participants that had been interviewed in all waves and were aged 50 or older in the 8th wave. A total of 58.7% of the sample were women and 41.3% were men, the mean age was 69.68 (*SD* = 8.95). Most of the sample reported living with their partner (69.1%), being retired (78.7%), having a higher secondary education (44.3%) and meeting ends without difficulties (59.2%). We describe the proportion of people by country in Table 1 following the European classification based on the United Nations Statistics Division criteria (2018) employed in recent studies (Georgieva et al. 2021).

**Table 1:** Number of participants from each country

	N (%)		N (%)
*Occidental*	*6987 (27.6%)*	*Meridional/South*	*9199 (29.0%)*
Austria	1196 (3.8%)	Croatia	1049 (3.3%)
Belgium	1540 (4.9%)	Greece	2542 (8.0%)
France	1556 (4.9%)	Italy	1746 (5.5%)
Germany	1735 (5.5%)	Malta	598 (1.9%)
Luxemburg	674 (2.1%)	Slovenia	1997 (6.3%)
Netherlands	468 (1.5%)	Spain	968 (3.1%)
Switzerland	1553 (4.9%)	Cyprus	299 (0.9%)
*Septentrional/North*	*7044 (22.2%)*	*Oriental/East*	*6062 (19.1%)*
Denmark	1144 (3.6%)	Bulgaria	554 (1.7%)
Estonia	2477 (7.8%)	Czech Republic	1616 (5.1%)
Finland	906 (2.9%)	Hungary	445 (1.4%)
Latvia	648 (2.0%)	Poland	1552 (4.9%)
Lithuania	1052 (3.3%)	Romania	1085 (3.4%)
Sweden	817 (2.6%)	Slovakia	810 (2.6%)
Israel	640 (2.0%)		

### Dependent variables

To assess the feelings of loneliness, we examined a single-item outcome of loneliness (“How much of the time do you feel lonely?”) with response options being "Often," "Some of the time," and "Hardly ever or never”. This measure was administered before the COVID-19 pandemic during regular Wave 8 (2019–2020) (Lonely T0) and during the pandemic in COVID Survey 1 (Lonely T1) and 2 (Lonely T2). Due to the low proportion of respondents who selected "Often" (less than 8% in each wave), we combined this category with "Some of the time" to create a binary measure. A score of 0 indicated no loneliness, while a score of 1 indicated some degree of loneliness.

### Independent

The main explanatory variable, voluntary or charity work participation, was measured across all three waves. In wave 8, participants were asked if they had done any voluntary work in the last twelve months (Volunteer T0), in COVID Survey 1 (Volunteer T1), they were asked if they had volunteered since the outbreak, and in COVID Survey 2 (Volunteer T2), they were asked if they had volunteered during the last three months. A score of 0 indicated no participation, while a score of 1 indicated yes. To study the pattern of volunteering, we created several dummy variables, including people who only participated in the eighth wave (Volunteer only T0), only participated in the COVID 1 survey (Volunteer only T1), only participated in the COVID 2 survey (Volunteer only T2), participated in volunteer activities in the first two time periods (Volunteer T0 T1), in the pre-pandemic period and in the second pandemic period (Volunteer T0 T2), during both pandemic periods (Volunteer T1 T2), and in all waves (Volunteer at all times), and we compared these dummy variables with the reference group that did not volunteer in any wave.

### Control variables

The analysis also considered some sociodemographic characteristics: age, treated as a continuous variable, gender (reference = men), living with partner (reference = living without partner), employment situation (reference = no retired), country of residence (introduced as dummy variables for each country with Greece as the reference country since it has the higher proportion of participants in the data). We also controlled for the respondent's ability to make ends meet (Ends meet) on a scale of 1 to 4 (1 = with great difficulty, 2 = with some difficulty, 3 = fairly easily, 4 = easily) (reference = great difficulty), education (coded using the SHARE-generated variable of the Internal Standard Classification of Education 1997 (ISCED97), ranging from 0 to 6). Mobility limitations were measured with the GALI indicator (reference = no limited). All these control variables were obtained from SHARE wave 8. In addition, we also controlled for a pandemic-specific variable, which was whether the respondent or anyone close to the respondent had tested positive for COVID-19 for each of the post-outbreak data collection points (reference = not positive for COVID). For the binary logistic regressions of loneliness, this variable was obtained from the COVID Survey 1 and COVID Survey 2. We included these control variables given that they were strongly correlated with volunteering activities or loneliness in old age. Individuals with a higher socioeconomic status (education and income) and not physical limited had a higher likelihood of engaging in volunteering activities (Mak and Fancourt 2022; Morrow-Howell 2010) and had a reduced risk of experiencing loneliness (Hawkley et al. 2008; Papa et al. 2019). Also, being a woman and living alone were related to reduced participation in volunteering activities (Mak and Fancourt. 2022; Niebuur et al. 2018) and greater feelings of loneliness (Vozikaki et al. 2018; Wei et al. 2022). Regarding European country differences, the rates of volunteering were lower in southern countries compared to the northern regions (Hansen et al. 2018) and the feelings of loneliness were higher (Vozikaki et al. 2018).

### Statistical analyses

We computed descriptive statistics (means, standard deviation, or frequency) for individuals who participated in volunteering activities before and during the pandemic. Also, we employed chi-square tests and V of Cramer effect sizes to compare categorical variables and an Analysis of Variance (ANOVA) and partial eta-squares effect sizes for the quantitative variables. Next, we estimated logistic regression models for loneliness outcomes. Given that loneliness was measured three times (once before and twice during the pandemic), we conducted three sets of models. For the first regression, "feeling lonely before the pandemic" (regular wave 2019–2020), we included one model with volunteering before the pandemic as an independent variable (M1). For the second regression, "feeling lonely during the first wave of the pandemic" (June and September 2020), we added volunteering during this pandemic period (M2). Finally, for the third regression, "feeling lonely during the second wave of the pandemic" (June and August 2021), we analyzed three models (M3, M4 and M5) which included volunteering before the pandemic, during the first wave of the pandemic, and during this second wave, respectively. Finally, we included a robustness check regression, also controlling for baseline loneliness presented in Tables 5 and 7. For all the variables included in the binary regressions, we presented the odds ratios (OR) or relative risk (RR) that provides information on the strength of the relationship between the variables, with an odds ratio significantly less than 1 indicating a lower likelihood of feeling lonely for the reference category, and an odds ratio significantly greater than 1 indicating a higher likelihood of feeling lonely compared to the reference category (Fernandes et al. 2020). The statistical analyses were performed using SPSS 28 and JASP 0.16.

## Results

### Volunteer characteristics

Table 2 displays the characteristics of individuals who participated in volunteering activities at any of these three points in time, at two of them, or who participated in all three points in time. First, descriptives are presented for people who volunteered during wave 0, 1 or 2, and then, descriptions of those who participated in more than one wave. Additional file 1 includes more detailed information about country distribution of volunteers over time (Supplemental Table 1).

**Table 2:** Characteristics of volunteers

	Volunteer T0	Volunteer T1	Volunteer T2	Volunteer T0–T1	Volunteer T0–T2	Volunteer T1–T2	Volunteer All times	*F* or χ^2^	η^2^ or V Cramer
Age	68.64 (8.04)	67.63 (7.33)	67.94 (7.31)	68.18 (7.64)	67.83 (7.23)	67.56 (7.12)	67.80 (6.87)	7.42***	0.01
Education	3.54 (1.36)	3.44 (1.44)	3.47 (1.42)	3.66 (1.36)	3.70 (1.34)	3.05 (1.46)	3.94 (1.28)	38.82***	0.04
Woman	59.0%	54.0%	52.6%	61.5%	56.3%	49.2%	46.9%	70.90***	0.10
Ends meet									
with great difficulty	5.3%	8.0%	6.7%	5.8%	3.9%	12.0%	1.8%	71.23***	0.12
with some difficulty	17.4%	18.0%	18.2%	15.5%	15.1%	17.7%	9.3%	74.90***	0.11
fairly easily	32.5%	31.1%	30.8%	31.6%	32.0%	32.5%	30.9%	5.69 ns	0.03
easily	44.8%	42.8%	44.3%	47.1%	49.0%	37.8%	58.0%	87.11***	0.11
Mobility limited	39.5%	40.8%	40.5%	42.4%	38.2%	45.8%	35.5%	15.05*	0.05
Living with partner	71.1%	72.2%	71.9%	73.2%	71.8%	71.0%	76.5%	14.37*	0.05
Retired	77.4%	77.7%	78.9%	77.3%	80.0%	80.6%	78.2%	7.47 ns	0.04
Occidental countries	41.0%	53.1%	54.6%	50.6%	56.6%	50.6%	65.5%	410.18***	0.25
Septentrional/North countries	18.3%	12.0%	12.6%	12.6%	10.0%	12.6%	5.7%	52.14***	0.09
Meridional/South countries	23.9%	20.0%	22.3%	22.5%	27.5%	22.5%	26.0%	522.85***	0.28
Oriental/East countries	15.2%	13.1%	8.4%	13.4%	5.4%	13.4%	2.7%	274.32***	0.20
Israel	1.6%	1.8%	2.2%	0.9%	0.5%	0.9%	0.0%	66.97***	0.10
N	4852	1585	2810	231	943	297	561		

T0 (Time 0 before pandemic, regular SHARE wave 8), T1 (time 1, SHARE COVID Survey 1), T2 (time 2, SHARE COVID Survey 2). * < 0.05, *** < 0.001.

In Table 2, we can observed how volunteering has evolved over time. Of the total study sample, 4852 individuals (15.6%) volunteered before the pandemic, while 1585 (5.0%) did so during the first wave of the COVID-19 pandemic, and 2810 (8.9%) volunteered during the second wave. Regarding those who volunteered twice, only 231 individuals (0.7%), a very small percentage, were able to do so during the first two time periods measured in this study, 943 individuals (3.0%) during the first and last time periods measured, and 297 (0.9%) during both pandemic time periods. Finally, 561 individuals (1.8%) participated in volunteering activities during all time periods, while the largest number, 24,503 individuals (77.4%) from the sample, never volunteered.

Throughout the waves, the profile of people who participated in volunteering activities remained relatively similar. Volunteers at different times have shown statistically significant differences in many characteristics but the effect sizes of these differences are small. However, it is important to note that the people who did not volunteer before the pandemic but started to volunteer in T1 and kept volunteering in T2 (Volunteer T1–T2) had a slightly different profile with higher rates of men, of people with difficulties to make ends meets, limited mobility and lower educational levels. Also, the profile of people that volunteered in all waves included individuals with a slightly higher educational level, a greater ability to make ends meet, those living with their partner and with fewer mobility limitations. Regarding the volunteers' country of residence at each moment in time, Occidental countries were the ones with the highest volunteer participation at all times, this is especially evident among the people who volunteer in all waves. Southern countries have the second highest number of volunteers, with a slight decrease in participation during T1 of the pandemic, while Northern countries are the ones that stopped volunteering the most since the beginning of the pandemic (before the pandemic, 18.3% of the people engaged in volunteering activities were from Northern countries, but this decreases by 12.0% during T1 and by 12.6% during T2). The involvement of people from Eastern countries seems to decrease during the second wave of the pandemic and finally, people from Israel seem to maintain levels of involvement in all waves, but there is no one from Israel involved in all waves.

### Feelings of loneliness and participation in volunteering

In Table 3, we can observe that only 50.4% of people who participated in volunteering activities before the pandemic continued during the first wave of the pandemic, and this increased to 54.1% during the second wave of the pandemic. Furthermore, only 30.8% of the people who volunteered during the first wave of the pandemic continued to do so in T2. We also describe the amount of participation in volunteering activities in T0 and during COVID-19. It is important to note that at T0 the question was about the frequency of volunteering in the last 12 months, in T1, the question was about the amount of volunteering since the outbreak, while at T2, it focused on the amount of volunteering compared to the first wave of the pandemic. We observed that during T0, most of the sample declare volunteering almost every week (39.7%), followed by almost every month (26.7%). In T1, similar percentages of volunteers reported increasing their participation frequency since COVID-19 (32.1%), decreasing it (32.2%), or maintaining the same levels (35.7%). However, the changes from T1 to T2 suggest that most volunteers (55.5%) maintained their participation frequency levels during the second time period after COVID-19, compared to T1. Regarding feelings of loneliness, there was a slight increase during the first and second wave of the COVID-19 pandemic.

**Table 3:** Volunteering activities and loneliness across T0, T1 and T2

	T0	T1	T2
Participated in volunteer T0		792 (50.4%)	1504 (54.1%)
Participated in volunteer T1			866 (30.8%)
*Amount of volunteering*			
Almost every day	600 (12.4%)	–	–
Almost every week	1927 (39.7%)	–	–
Almost every month	1294 (26.7%)	–	–
Less often	1029 (21.2%)	–	–
*Amount of volunteering -*			
More often	–	509 (32.1%)	657 (23.4%)
About the same	–	566 (35.7%)	1557 (55.5%)
Less often	–	510 (32.2%)	591 (21.1%)
Loneliness	8626 (27.7%)	8993 (28.5%)	9931 (31.4%)

T0 (Time 0 before pandemic, regular SHARE wave 8), T1 (time 1, SHARE COVID Survey 1), T2 (time 2, SHARE COVID Survey 2)

As for the question, whether volunteering is associated with a lower likelihood of experiencing feelings of loneliness, Table 4 presents our analysis of volunteering and other predictors of loneliness before the pandemic and during two different time periods of the pandemic. Additional file 1 includes robustness check analysis controlling for baseline loneliness (Supplemental Table 2).

**Table 4:** Odds ratio of the variables from logistic regressions

	Lonely T0	Lonely T1		Lonely T2		
	(M1)	(M2)	(M3)	(M4)	(M5)	(M6)
Intercept	0.420***	0.431***	0.430***	0.487***	0.487***	0.487***
Volunteer T0	0.77***	0.86**		0.85**		
Volunteer T1			0.90		0.96	
Volunteer T2						0.73***
Age	1.00*	1.0***	1.02***	1.02***	1.02***	1.02***
Woman	1.01	1.42***	1.42***	1.52***	1.52***	1.52***
Education	0.94***	0.99	1.00	0.99	0.99	0.99
Ends meet (Ref = great difficulty)						
some difficulty	0.65***	0.86**	0.85**	0.84**	0.84**	0.84**
fairly easily	0.52***	0.71***	0.71***	0.69***	0.68***	0.68***
easily	0.41***	0.62***	0.62***	0.62***	0.61***	0.62***
Living with partner	0.36***	0.27***	0.27***	0.32***	0.32***	0.32***
Mobility limited	1.65***	1.42***	1.43***	1.48***	1.49***	1.48***
Retired	1.23***	0.98	0.99	0.96	0.95	0.96
Test positive COVID		0.99	1.05	1.00	1.00	1.01
Country (Ref = Greece)						
Israel	0.98	0.36***	0.35***	0.41***	0.40***	0.41***
*Occidental*						
Austria	0.27***	0.19***	0.18***	0.14***	0.13***	0.14***
Belgium	0.59***	0.39***	0.38***	0.26***	0.26***	0.27***
France	0.74**	0.43***	0.42***	0.36***	0.35***	0.36***
Germany	0.39***	0.31***	0.31***	0.24***	0.23***	0.24***
Luxembourg	0.44***	0.38***	0.37***	0.29***	0.28***	0.29***
Netherlands	0.59**	0.30***	0.29***	0.17***	0.16***	0.17***
Switzerland	0.81*	0.22***	0.22***	0.16***	0.16***	0.17***
*North*						
Denmark	0.24***	0.18***	0.17***	0.11***	0.10***	0.11***
Estonia	0.43***	0.26***	0.26***	0.25***	0.25***	0.25***
Finland	0.73**	0.27***	0.27***	0.23***	0.23***	0.23***
Latvia	0.63***	0.49***	0.52***	0.50***	0.49***	0.50***
Lithuania	0.50***	0.30***	0.30***	0.29***	0.29***	0.29***
Sweden	0.55***	0.37***	0.37***	0.25***	0.25***	0.25***
*South*						
Croatia	0.85	0.57***	0.57***	0.62***	0.61***	0.61***
Italy	1.31**	0.84	0.82	0.81*	0.80*	0.80*
Malta	0.67*	0.49***	0.49***	0.31***	0.30***	0.31***
Slovenia	0.36***	0.23***	0.23***	0.21***	0.21***	0.21***
Spain	0.53***	0.32***	0.31***	0.27***	0.26***	0.27***
Cyprus	0.92	0.53***	0.52***	0.50***	0.49***	0.49***
*East*						
Bulgaria	0.83	0.41***	0.40***	0.58***	0.56***	0.59***
Czech Republic	0.49***	0.33***	0.33***	0.31***	0.30***	0.30***
Hungary	0.46***	0.27***	0.27***	0.36***	0.36***	0.36***
Poland	0.57***	0.36***	0.36***	0.31***	0.31***	0.30***
Romania	0.73**	0.42***	0.42***	0.34***	0.34***	0.34***
Slovakia	1.20	0.83	0.84	0.68**	0.68**	0.68**
N	18,065	17,956	17,954	17,882	17,926	17,923

This table includes full results from logistic regression models, T0 (Time 0 before pandemic, regular SHARE wave 8), T1 (time 1, SHARE COVID Survey 1), T2 (time 2, SHARE COVID Survey 2). Bold indicates statistical significance: *p < 0.05; **p < 0.01; ***p < 0.001

Regarding the association between volunteering and feelings of loneliness, Model 1, Model 2, and Model 4 indicate that the people who participated in volunteering activities prior to the pandemic had a lower risk of feeling lonely in T0, T1 and T2, respectively, compared to those who did not engage in volunteering activities. However, Model 3 and Model 5 reveal that volunteering during the early pandemic period was not significantly related to loneliness in the first months of the pandemic (T1), nor a year after (T2). However, Model 6 demonstrates that individuals who volunteered during the second pandemic period were less likely to experience loneliness during that time.

Throughout all time periods, being older and having mobility limitations were statistically associated with a higher likelihood of feeling lonely, while living with a partner and having a better financial situation were statistically associated with a lower likelihood of feeling lonely. Women were more likely to feel lonely, but this gender effect was only significant during the pandemic, conversely, being retired only was statistically related with a higher likelihood of feeling lonely before the pandemic. Educational level and testing positive for COVID-19 were not associated with a higher likelihood of feeling lonely at any time. Lastly, almost all the countries showed less likelihood of experiencing loneliness in each time period compared to the country of reference Greece, with higher differences among Greece and the North and Occidental countries.

Furthermore, we conducted the same analysis, adding the loneliness baseline measure as a control, which can be found in Table 5. From the results, we observed that even after controlling for baseline loneliness levels, volunteering during the time period prior to the pandemic was still significantly associated with a lower likelihood of feeling lonely during the second wave of the pandemic, the odds ratio (OR) was 0.89. In addition, being engaged in volunteering activities during the second wave of the pandemic was also significantly associated with a lower likelihood of feeling lonely (OR = 0.73).

**Table 5:** Robustness check controlling for baseline loneliness

	**Lonely T1**		**Lonely T2**		
	**(M2)**	**(M3)**	**(M4)**	**(M5)**	**(M6)**
Intercept	0.43***	0.43***	0.49***	0.48***	0.48***
Loneliness baseline	3.55***	3.55***	3.28***	3.28***	3.29***
Volunteer T0	0.91		0.89*		
Volunteer T1		0.887		0.95	
Volunteer T2					0.73***
N	17,915	17,929	17,841	17,850	17,847

All control variables described in the main documents are included in the models and displayed in a Supplementary Table. Loneliness baseline was added as additional control variable for robustness check. Bold indicates statistical significance: *p < 0.05; **p < 0.01; ***p < 0.001

### Patterns of volunteering participation and feelings of loneliness during the pandemic

Table 6 displays the results of volunteering participation during the pandemic and explored if this participation in different moments was related to feelings of loneliness. In the first regression analysis of loneliness feelings during the first pandemic period, we observed that people who had volunteered before the pandemic had a lower likelihood of feeling lonely during the first pandemic wave compared with those who did not volunteer at any time (OR = 0.86).

**Table 6:** Odds ratio of volunteer participation across T0, T1 and T2

Ref. No volunteer at any time	Lonely T1	Lonely T2
Intercept	0.43***	0.49***
Volunteer T0	0.86**	0.93
Volunteer T1	0.90	1.11
Volunteer T0 T1	0.83	0.88
Volunteer T2		0.75**
Volunteer T0 T2		0.61***
Volunteer T1 T2		0.97
Volunteer at all times		0.69*
Age	1.02***	1.02***
Woman	1.42***	1.51***
Education	0.99	0.99
*Ends meet (Ref* = *great difficulty)*		
Some difficulty	0.86*	0.85*
Fairly easily	0.71***	0.69***
Easily	0.62***	0.62***
Living with partner	0.27***	0.32***
Mobility limited	1.42***	1.48***
Retired	0.99***	0.96
Test positive COVID	0.99	0.98
N	17,954	17,908

All control variables described in the main documents are included in the models, OR of country dummy variables are not shown for clarity but are included in a Supplementary Table. T1 (time 1, SHARE COVID Survey 1), T2 (time 2, SHARE COVID Survey 2). Bold indicates statistical significance: * *p* < 0.05; **p < 0.01; ***p < 0.001

During the second pandemic wave, the results were more complicated, as data on three waves were combined. We were able to observe that participation in volunteering activities only during the second pandemic period, before and during the second pandemic period, and during all three periods was significantly and inversely related to feelings of loneliness during the second pandemic period compared to not participating in any volunteering activities at any time OR = 0.75, OR = 0.61, and OR = 0.69, respectively.

The robust analysis presented in Table 7, which includes the same variables as before, as well as the loneliness baseline measure, illustrates how the significance of participating in volunteering activities on feelings of loneliness compared to not participating at any time was reduced during the first wave of the pandemic. However, the effects of volunteering during certain time periods remained statistically significant compared to those who did not volunteer during any measured time and volunteering was related to a lower likelihood of feeling lonely during the second wave of the pandemic. OR = 0.73, for those who volunteered during the second pandemic wave, OR = 0.63, for those who participated after the pandemic and during the second time, and OR = 0.70, for those who volunteered during all time periods.

**Table 7:** Robustness check controlling for baseline loneliness

	Lonely T1	Lonely T2
Intercept	0.43***	0.49***
Loneliness baseline	3.55***	3.30***
*Ref* = *No volunteer at any time*		
Volunteer T0	0.84	0.98
Volunteer T1	0.91	1.10
Volunteer T0 T1	0.88	0.86
Volunteer T2		0.73**
Volunteer T0 T2		0.63***
Volunteer T1 T2		0.96
Volunteer at all times		0.70*
N	17,913	17,866

All control variables described in the main documents are included in all models but are included in a Supplementary Table. This is an additional control variable added as a robustness check. Bold indicates statistical significance: *p < 0.05; **p < 0.01; ***p < 0.001

## Discussion

This study examines the characteristics of the volunteer participation and the relationship between volunteer participation and feelings of loneliness in the aftermath of the COVID-19 outbreak. As our main objective of study, we investigated the relationship between volunteering and feelings of loneliness. Our results indicate that participation in volunteering activities before the pandemic was significantly associated with a lower risk of loneliness during all measured time periods, highlighting its potential as a strategy to increase resilience toward loneliness in times of crisis such as the COVID-19 pandemic. However, volunteering activities during the first wave of the pandemic did not show a significant association with loneliness. This seems surprising when compared to the results of other studies that have shown various individual benefits for older volunteers who were able to continue volunteering during the pandemic lockdown.

Also, it is important to note that the people who volunteer before the pandemic, show different characteristics that may help to explain these results. For example, people who volunteer before the pandemic were older, have higher levels of education, less economic difficulties, and less mobility limitations than those who started volunteering after the COVID-19. Some of these characteristics, especially higher socioeconomic status, and good health, has been related to decreased risk of loneliness and increased likelihood of volunteering (Carr et al. 2018; Morrow-Howell 2010). Maybe this “usual” profile of volunteer is more beneficiated by the action of volunteer because they have more continuity in this activity as a result of their higher commitment and alignment with altruist values. Volunteering seems to be more strongly associated with less loneliness when the continuity and intensity of volunteering is higher (Akhter-Khan et al. 2022; Windsor et al. 2008). People in our study who volunteered prior to the COVID-19 exhibited more consistent participation in the following years (50.4% in T1 and 54.1% in T2), compared to those who began volunteering during the first year of the pandemic (only 30.8% continued in the subsequent year).

We also need to consider that while the people who participated in volunteering activities before the pandemic did so out of personal interest, during the pandemic they may have felt compelled to do so. For instance, Chan et al. (2021) found that volunteering was associated with fewer symptoms of depression and anxiety. However, their results may be influenced by the fact that they used a convenience sample of people who had already started volunteering in a program prior to COVID-19 and maybe these individuals had a higher motivation for initiating and sustaining their volunteering activities. Also, existing literature suggests that the protective effects of volunteering stem from the rewarding experiences it provides and not just from the act of volunteering itself. For example, Hansen et al. (2018) and Andersen et al. (2022) found that people in Denmark felt coerced to participate in volunteering activities during the COVID-19 lockdown.

Another possible explanation that we need to consider is that many volunteering opportunities were cancelled or limited during this time, and individuals may have experienced a sense of helplessness or inability to contribute to their communities in the way they typically would have through volunteering (Henning-Smith 2020; Miller 2020). The volunteering activities also changed due to the COVID-19 lockdown. Some volunteering activities specifically initiated due to COVID-19, such as distributing face masks to people in need or providing pandemic prevention materials like sanitizers, did not appear to prevent feelings of anxiety and depression in older adults (Chan et al. 2021), so it is possible that these activities did not prevent feelings of loneliness either. In addition, many of the activities that used to be done face-to-face moved online (Lachance 2021), and although these new forms of volunteering during the pandemic may have generated new opportunities, greater interest, and better attitudes of older adults toward virtual volunteering (Sun et al. 2021), it is likely that people needed time to adapt to the changes.

The results also demonstrate that volunteering during the second wave of the pandemic was significantly associated with a lower likelihood of feeling lonely during that time. Even after controlling for baseline levels of loneliness, volunteering activities prior to the pandemic and during the second time were still significantly associated with a lower likelihood of feeling lonely during the second wave of the pandemic. Taking into account that our first period of time, measured before the pandemic, was collected between October 2019 and March 2020 and the second COVID-19 time was collected between June and August 2021, our findings are in line with Biddle and Grey, 2021 that compared a group of volunteers in late 2019 and in April 2021 and concluded that those who stopped volunteering were far more likely to say that they felt lonely at least some of the time than those who also volunteer in April 2021.

Regarding the patterns in volunteer participation during the pandemic and its relationship with feelings of loneliness. The results suggest that individuals who participated in volunteering activities only during the second wave of the pandemic, before and during the second wave, as well as those who participated in all waves, had a significantly lower likelihood of feeling lonely during the second wave. This highlights the potential benefits of continuing to engage in volunteering activities (Chan et al. 2021; Sin et al. 2021), even if they are not possible during the earlier stages of a crisis.

This study has some limitations that should be acknowledged. The main limitation is in regard to the measure of volunteering used in this study. We assessed whether individuals were involved in volunteering activities or not, and although we were able to analyze this variable across different time periods and the changes in participation, we did not consider other important factors such as the type of volunteering activity in which the people were involved or their motivations for participating. These variables could be important when assessing the impact of volunteering during the COVID-19 pandemic (Principi et al. 2022).

In addition, we found some interesting results that should be addressed in greater detail. For instance, in our sample, people who volunteered after the onset of the pandemic had greater mobility limitations compared to volunteers who started before COVID-19. In a study by Mark and Fancourt (2022), it was also found that people with a diagnosed physical illness or disability were more likely to engage in social action volunteering during COVID-19, and this could indicate a desire to collaborate, but in activities that can be done from one's own home, such as participating in Internet searches. Future studies should investigate how best to promote this type of activity among older people with functional limitations who want to participate. Also, the percentage of older people volunteering increased in Israel and Western and Southern European countries, however participation in Northern and Eastern countries decreased after the start of COVID-19. In general, participation in volunteering activities is higher in Northern countries than in Southern countries (Lee 2022b). Future studies should try to understand these changes in the attitudes toward volunteering during the COVID-19 pandemic.

Moreover, it should be noted that while the overall study sample is large, certain subgroups of volunteers analyzed have small sample sizes (e.g., only 297 respondents participated during the two waves of the pandemic) and this may result in some of the regression coefficients being non-significant. There are also some additional limitations, for example, we did not consider the potential differential effects of volunteering based on the geographical area in which individuals live. Volunteering may have a greater impact in rural areas where other services are limited (Colibaba et al. 2021; Davies et al. 2018), and this could be an important factor to consider in future research. Although our study controlled for country in all analyses, we did not explore potential differences between countries. Various European countries implemented different measures to address the COVID-19 pandemic (Engler et al. 2021), and the impact of volunteering on feelings of social recognition and health may vary depending on the level of government support for such activities (Hansen et al. 2018). These differences were not explored in-depth and could be a valuable path for future research.

Nevertheless, despite the limitations, our research has the strength of having been done with a very large cross-national sample of the older adult population, with baseline pre-pandemic data and single post-pandemic outbreak measures collected over two time periods during the COVID-19 pandemic. These results have important policy implications, given that they indicate that, through volunteering, older people can counteract feelings of loneliness even in difficult times of self-isolation and physical detachment such as the times of the COVID-19 pandemic. It is important that in emergency situations involving older individuals, policy makers not only consider them as recipients of health and social care, but also as useful providers of help in the community. The encouragement of volunteering among older people can be considered a useful strategy to avoid the feelings of loneliness during future emergency situations such as the COVID-19 outbreak.

## Conclusions

In conclusion, this study sheds light on the impact of the COVID-19 pandemic on volunteering activities and the relationship between volunteering and feelings of loneliness among older adults from Europe and Israel. Our main findings indicate that volunteering before the pandemic was associated with a lower likelihood of feeling lonely at all measured time periods. However, volunteering during the first wave of the pandemic did not show a significant association with loneliness. This effect may have several explanations such as the potential coercion felt by individuals to participate in volunteering activities during the pandemic, limited opportunities for volunteering, which made it difficult for older people to contribute in the way they would normally have done, and the need for adaptation to the changes brought about by the pandemic. However, volunteering during the second wave of the pandemic was significantly associated with a lower likelihood of feeling lonely during that time. This research provides important policy implications as it shows that through volunteering, older people can manage feelings of loneliness even in difficult times such as COVID-19.

### Supplementary Information

Below is the link to the electronic supplementary material.

**Additional file 1.** Suplementary analyses.

## Data Availability

The data that support the findings of this study are available at the SHARE Research Data Center to the entire research community free of charge (www.share-project.org). Restrictions apply to the availability of these data, which were used under license for the current study, which were used under license for the current study, and so are not publicly available. Data are however available from the authors upon reasonable request and with permission of the SHARE Project (http://www.share-project.org/data-access/user-registration.html?L =).

## References

[CR1] Akhter-Khan SC, Hofmann V, Warncke M, Tamimi N, Mayston R, Prina MA (2022). Caregiving, volunteering, and loneliness in middle-aged and older adults: a systematic review. Aging Ment Health.

[CR2] Andersen D, Toubøl J, Kirkegaard S, Bang Carlsen H (2022). Imposed volunteering: gender and caring responsibilities during the COVID-19 lockdown. Sociological Review.

[CR3] Arpino B, Mair CA, Quashie NT, Antczak R (2022). Loneliness before and during the COVID-19 pandemic—are unpartnered and childless older adults at higher risk?. Eur J Ageing.

[CR4] Atzendorf J, Gruber S (2022). Depression and loneliness of older adults in Europe and Israel after the first wave of covid-19. Eur J Ageing.

[CR5] Biddle N, Gray M (2021). Volunteering during the first year of the COVID-19 pandemic.

[CR6] Börsch-Supan A, Brandt M, Hunkler C, Kneip T, Korbmacher J, Malter F, Schaan B, Stuck S, Zuber S (2013). Data resource profile: the survey of health, ageing and retirement in europe (SHARE). Int J Epidemiol.

[CR7] Börsch-Supan A (2022a) *Survey of Health, Ageing and Retirement in Europe (SHARE) Wave 8. Release version: 8.0.0.* SHARE-ERIC. Data set. 10.6103/SHARE.w8.800

[CR8] Börsch-Supan A (2022b) Survey of Health, Ageing and Retirement in Europe (SHARE) Wave 8. COVID-19 Survey 1. Release version: 8.0.0. SHARE-ERIC. Data set. 10.6103/SHARE.w8ca.800

[CR9] Börsch-Supan A (2022c) Survey of Health, Ageing and Retirement in Europe (SHARE) Wave 9. COVID-19 Survey 2. Release version: 8.0.0. SHARE-ERIC. Data set. 10.6103/SHARE.w9ca.800

[CR10] Boss L, Kang D, Branson S (2015). Loneliness and cognitive function in the older adult: a systematic review. Int Psychogeriatr.

[CR11] Bu F, Steptoe A, Fancourt D (2020). Who is lonely in lockdown? Cross-cohort analyses of predictors of loneliness before and during the COVID-19 pandemic. Public Health.

[CR12] Butrica BA, Johnson RW, Zedlewski SR (2009). Volunteer dynamics of older Americans. J Gerontol B Psychol Sci Soc Sci.

[CR13] Cacioppo JT, Hawkley LC (2009). Perceived social isolation and cognition. Trends Cognit Sci.

[CR14] Callow M (2004). Identifying promotional appeals for targeting potential volunteers: an exploratory study on volunteering motives among retirees. Int J Nonprofit Volunt Sect Mark.

[CR15] Campagne DM (2019). Stress and perceived social isolation (loneliness). Arch Gerontol Geriatr.

[CR16] Carr DC, Kail BL, Matz-Costa C, Shavit YZ (2018). Does becoming a volunteer attenuate loneliness among recently widowed older adults?. J Gerontol Ser B.

[CR17] Chambre SM (1984). Is volunteering a substitute for role loss in old age? An empirical test of activity theory. Gerontologist.

[CR18] Chan W, Chui CHK, Cheung JCS, Lum TYS, Lu S (2021). Associations between volunteering and mental health during COVID-19 among Chinese older adults. J Gerontol Soc Work.

[CR19] Chatzi G, Di Gessa G, Nazroo J (2020) Changes in older people's experiences of providing care and of volunteering during the COVID-19 pandemic

[CR20] Cho J, Xiang X (2022). The relationship between volunteering and the occurrence of loneliness among older adults: a longitudinal study with 12 years of follow-up. J Gerontol Soc Work.

[CR21] Colibaba A, Skinner MW, Russell E (2021). Rural aging during COVID-19: a case study of older voluntarism. Can J Aging.

[CR22] Courtin E, Knapp M (2017). Social isolation, loneliness and health in old age: a scoping review. Health Soc Care Community.

[CR23] Coyle CE, Dugan E (2012). Social isolation, loneliness and health among older adults. J Aging Health.

[CR24] Curvers N, Pavlova M, Hajema K, Groot W, Angeli F (2018). Social participation among older adults (55+): results of a survey in the region of South Limburg in the Netherlands. Health Soc Care Community.

[CR25] Davies A, Lockstone-Binney L, Holmes K (2018). Who are the future volunteers in rural places? Understanding the demographic background characteristics of non-retired rural volunteers, why they volunteer and their future migration intentions. J Rural Stud.

[CR26] De Jong-Gierveld J, Havens B (2004). Cross-national comparisons of social isolation and loneliness: introduction and overview’. Canadian Journal on Aging Revue Canadienne Du Vieillissement.

[CR27] Domènech-Abella J, Mundó J, Switsers L, Van Tilburg T, Fernández D, Aznar-Lou I (2021). Social network size, loneliness, physical functioning and depressive symptoms among older adults: examining reciprocal associations in four waves of the Longitudinal Aging Study Amsterdam (LASA). Int J Geriatr Psychiatry.

[CR28] Dury S, De Donder L, De Witte N, Buffel T, Jacquet W, Verte D (2015). To volunteer or not: The influence of individual characteristics, resources, and social factors on the likelihood of volunteering by older adults. Nonprofit Volunt Sect Q.

[CR29] Engler S, Brunner P, Loviat R, Abou-Chadi T, Leemann L, Glaser A, Kübler D (2021). Democracy in times of the pandemic: explaining the variation of COVID-19 policies across European democracies. West Eur Polit.

[CR30] Ernst JM, Cacioppo JT (1999). Lonely hearts: psychological perspectives on loneliness. Appl Prev Psychol.

[CR31] Fernandes AAT, Filho DBF, da Rocha EC, da Silva NW (2020). Read this paper if you want to learn logistic regression. Revista De Sociologia e Politica.

[CR32] Georgieva S, Carbonell Á, Martínez-Gregorio S, Alberola S, Oliver A (2021) Quality of life predictors in European aging population: A multigroup model. *Anuario de *Psicología/The UB Journal of Psychology, 51(3)

[CR33] Gerst-Emerson K, Jayawardhana J (2015). Loneliness as a public health issue: the impact of loneliness on health care utilization among older adults. Am J Public Health.

[CR34] Grotz J, Dyson S, Birt L (2020). Pandemic policy making: the health and wellbeing effects of the cessation of volunteering on older adults during the COVID-19 pandemic. Qual Ageing Older Adults.

[CR35] Hansen T, Aartsen M, Slagsvold B, Deindl C (2018). Dynamics of volunteering and life satisfaction in midlife and old age: findings from 12 European countries. Soc Sci.

[CR36] Hawkley LC, Cacioppo JT (2010). Loneliness matters: a theoretical and empirical review of consequences and mechanisms. Ann Behav Med.

[CR37] Hawkley LC, Hughes ME, Waite LJ, Masi CM, Thisted RA, Cacioppo JT (2008). From social structural factors to perceptions of relationship quality and loneliness: the Chicago health, aging, and social relations study. J Gerontol B Psychol Sci Soc Sci.

[CR38] Hawkley LC, Thisted RA, Cacioppo JT (2009). Loneliness predicts reduced physical activity: cross-sectional & longitudinal analyses. Health Psychol.

[CR39] Henning-Smith C (2020). The unique impact of COVID-19 on older adults in rural areas. J Aging Soc Policy.

[CR40] Kim ES, Whillans AV, Lee MT, Chen Y, Vander Weele TJ (2020). Volunteering and subsequent health and well-being in older adults: an outcome-wide longitudinal approach. Am J Prev Med.

[CR41] Kojima G, Taniguchi Y, Aoyama R, Tanabe M (2022). Associations between loneliness and physical frailty in community-dwelling older adults: a systematic review and meta-analysis. Ageing Res Rev.

[CR42] Krendl AC, Perry BL (2021). The impact of sheltering in place during the COVID-19 pandemic on older adults’ social and mental well-being. J Gerontol Ser B.

[CR43] Kristensen SM, Urke HB, Larsen TB, Danielsen AG (2022). Hello darkness, my old friend: moderating a random intercept cross-lagged panel model of loneliness and symptoms of anxiety and depression. Res Child Adolesc Psychopathol.

[CR44] Lachance EL (2021). COVID-19 and its impact on volunteering: moving towards virtual volunteering. Leis Sci.

[CR45] Lee S (2022). Volunteering and loneliness in older adults: a parallel mediation model. Aging Ment Health.

[CR46] Lee S (2022). The volunteer and charity work of European older adults: findings from SHARE. J Nonprofit Public Sect Mark.

[CR47] Lee S (2023). Loneliness, volunteering, and quality of life in European older adults. Act Adapt Aging.

[CR48] Mak HW, Fancourt D (2022). Predictors of engaging in voluntary work during the COVID-19 pandemic: analyses of data from 31,890 adults in the UK. Perspect Public Health.

[CR49] Miller EA (2020). Protecting and improving the lives of older adults in the COVID-19 era. J Aging Soc Policy.

[CR50] Morrow-Howell N (2010). Volunteering in later life: Research frontiers. J Gerontol B Psychol Sci Soc Sci.

[CR51] Niebuur J, Van Lente L, Liefbroer AC, Steverink N, Smidt N (2018). Determinants of participation in voluntary work: a systematic review and meta-analysis of longitudinal cohort studies. BMC Public Health.

[CR52] Niedzwiedz CL, Richardson EA, Tunstall H, Shortt NK, Mitchell RJ, Pearce JR (2016). The relationship between wealth and loneliness among older people across Europe: Is social participation protective?. Prev Med.

[CR53] Papa R, Cutuli G, Principi A, Scherer S (2019). Health and volunteering in Europe: a longitudinal study. Res Aging.

[CR54] Posch M, Bauer P, Posch A, König F (2020). Analysis of Austrian COVID-19 deaths by age and sex. Wien Klin Wochenschr.

[CR55] Principi A, Lucantoni D, Quattrini S, Di Rosa M, Socci M (2022). Changes in volunteering of older adults in the time of the COVID-19 pandemic: the role of motivations. Int J Environ Res Public Health.

[CR56] Sin NL, Klaiber P, Wen JH, De Longis A (2021). Helping amid the pandemic: daily affective and social implications of COVID-19-related prosocial activities. Gerontologist.

[CR57] Singh A, Misra N (2009). Loneliness, depression and sociability in old age. Ind Psychiatry J.

[CR58] Sirven N, Berchet C, Litwin H (2015). Social participation and health: a cross-country investigation among older Europeans. Soc Cap Health Resour Later Life Relevance Context.

[CR59] Snyder M, Clary EG, Stukas AA, Maio GR, Olson JM (2000). The functional approach to volunteerism. Why we evaluate: Functions of attitudes.

[CR60] Stukas AA, Hoye R, Nicholson M, Brown KM, Aisbett L (2016). Motivations to volunteer and their associations with volunteers’ well-being. Nonprofit Volunt Sect Q.

[CR61] Sun PC, Morrow-Howell N, Pawloski E, Helbach A (2021). Older adults’ attitudes toward virtual volunteering during the COVID-19 pandemic. J Appl Gerontol.

[CR62] Sundström M, Blomqvist K, Edberg AK (2021). Being a volunteer encountering older people’s loneliness and existential loneliness: alleviating loneliness for others and oneself. Scand J Caring Sci.

[CR63] Taniguchi H (2012). The determinants of formal and informal volunteering: Evidence from the American time use survey. Volunt Int J Volunt Nonprofit Organ.

[CR64] Tesch-Römer C, Huxhold O (2019). Social isolation and loneliness in old age. Oxford Res Encycl Subj Develop Psychol.

[CR65] United Nations Statistics Division (2018) Statistics Division M49 Standard. https://unstats.un.org/unsd/methodolo-gy/m49/

[CR66] Valtorta NK, Kanaan M, Gilbody S, Hanratty B (2018). Loneliness, social isolation and risk of cardiovascular disease in the English Longitudinal Study of Ageing. Eur J Prev Cardiol.

[CR67] Victor C, Scambler S, Bond J, Bowling A (2000). Being alone in later life: loneliness, social isolation and living alone. Rev Clin Gerontol.

[CR68] Vozikaki M, Papadaki A, Linardakis M, Philalithis A (2018). Loneliness among older European adults: results from the survey of health, aging and retirement in Europe. J Public Health.

[CR69] Wei K, Liu Y, Yang J, Gu N, Cao X, Zhao X, Jiang L, Li C (2022). Living arrangement modifies the associations of loneliness with adverse health outcomes in older adults: evidence from the CLHLS. BMC Geriatr.

[CR70] Wilson J, Musick M (1997). Who cares? Toward an integrated theory of volunteer work. Am Sociol Rev.

[CR71] Windsor TD, Anstey KJ, Rodgers B (2008). Volunteering and psychological well-being among young-old adults: How much is too much?. Gerontologist.

