# Supplementary material for: Senior volunteers: addressing loneliness in times of COVID-19
Source: Eur J Ageing. 2023 Oct 20;20(1):40. doi: 10.1007/s10433-023-00788-5 (PMC10589161; doi:10.1007/s10433-023-00788-5)
Supplement: Supplementary file 1 — Additional file 1. Suplementary analyses. [file 10433_2023_788_MOESM1_ESM.docx]

Supplemental Tables

Supplemental Table 1. Country distribution of volunteers over time

|  | **Volunteer**  **T0** | **Volunteer**  **T1** | **Volunteer**  **T2** | **Volunteer**  **T0 - T1** | **Volunteer**  **T0 – T2** | **Volunteer**  **T1 – T2** | **Volunteer**  **All times** |
| --- | --- | --- | --- | --- | --- | --- | --- |
| Israel | 12.3% | 1.8% | 2.2% | 0.9% | 1.1% | 6.7% | 1.1% |
| **Occidental** |  |  |  |  |  |  |  |
| Austria | 22.7% | 6.6% | 6.9% | 9.1% | 7.5% | 6.1% | 9.1% |
| Belgium | 22.2% | 8.6% | 8.8% | 8.7% | 0.5% | 10.4% | 0.4% |
| France | 27.9% | 10.9% | 8.6% | 13.9% | 13.5% | 4.7% | 11.9% |
| Germany | 26.5% | 9.1% | 9.8% | 10.8% | 14.2% | 3.4% | 16.9% |
| Luxembourg | 26.1% | 3.1% | 3.5% | 3.0% | 0.5% | 1.0% | 4.3% |
| Netherlands | 47.4% | 5.4% | 4.9% | 4.3% | 6.3% | 1.0% | 11.6% |
| Switzerland | 7.4% | 9.4% | 12.0% | 0.9% | 8.7% | 31.3% | 7.8% |
| **North** |  |  |  |  |  |  |  |
| Denmark | 35.7% | 6.8% | 8.5% | 8.7% | 0.7% | 4.7% | 0.5% |
| Estonia | 11.0% | 2.0% | 2.6% | 5.6% | 1.0% | 0.0% | 0.2% |
| Finland | 28.1% | 6.1% | 5.3% | 0.9% | 5.1% | 5.7% | 8.2% |
| Latvia | 7.0% | 0.9% | 1.5% | 9.1% | 1.4% | 1.7% | 0.2% |
| Lithuania | 3.6% | 0.8% | 0.5% | 0.0% | 7.5% | 0.3% | 9.1% |
| Sweden | 18.2% | 3.5% | 4.0% | 2.2% | 4.1% | 3.4% | 3.7% |
| **South** |  |  |  |  |  |  |  |
| Croatia | 5.2% | 0.8% | 0.9% | 0.9% | 0.3% | 1.0% | 0.2% |
| Italy | 12.9% | 2.4% | 2.2% | 0.4% | 12.2% | 5.4% | 14.3% |
| Malta | 16.6% | 0.2% | 1.3% | 0.0% | 2.2% | 0.0% | 0.5% |
| Slovenia | 12.6% | 3.5% | 4.5% | 4.3% | 3.1% | 1.3% | 0.2% |
| Spain | 10.0% | 1.3% | 1.0% | 2.2% | 0.4% | 1.7% | 0.2% |
| Cyprus | 10.9% | 0.6% | 0.3% | 5.2% | 0.0% | 0.7% | 0.0% |
| Greece | 6.0% | 3.1% | 2.3% | 4.3% | 2.0% | 1.7% | 1.8% |
| **East** |  |  |  |  |  |  |  |
| Bulgaria | 26.6% | 1.1% | 3.6% | 0.4% | 14.2% | 1.7% | 16.9% |
| Czech Republic | 25.7% | 7.7% | 2.0% | 11.7% | 0.7% | 4.7% | 4.1% |
| Hungary | 5.9% | 0.8% | 0.6% | 0.4% | 5.6% | 0.0% | 1.8% |
| Poland | 4.7% | 1.5% | 1.4% | 0.9% | 5.7% | 1.3% | 0.7% |
| Romania | 4.3% | 0.9% | 0.3% | 0.0% | 0.3% | 0.0% | 0.2% |
| Slovakia | 5.8% | 1.1% | 0.5% | 0.0% | 0.3% | 0.0% | 0.2% |
| N | 4852 | 1585 | 2810 | 231 | 943 | 297 | 561 |
| *Note:* T0 (Time 0 before pandemic, regular SHARE wave 8), T1 (time 1, SHARE Covid Survey 1), T2 (time 2, SHARE Covid Survey 2). | | | | | | | |

Supplemental Table 2. Robustness check controlling for baseline loneliness including country dummies

|  | **Lonely T1** | | **Lonely T2** | | |
| --- | --- | --- | --- | --- | --- |
|  | **(M2)** | **(M3)** | **(M4)** | **(M5)** | **(M6)** |
| Intercept | **.43***** | **.43***** | **.49***** | **.48***** | **.48***** |
| Loneliness baseline | **3.55***** | **3.55***** | **3.28***** | **3.28***** | **3.29***** |
| Volunteer | .91 |  | **.89*** |  |  |
| Volunteer T1 |  | .887 |  | .95 |  |
| Volunteer T2 |  |  |  |  | **.73***** |
| Age | **1.02***** | **1.02***** | **1.02***** | **1.57***** | **1.02***** |
| Women | **1.46***** | **1.46***** | **1.57***** | **1.02***** | **1.56***** |
| Education | **1.01** | **1.01** | 1.01 | 1.01 | 1.01 |
| Ends meet (Ref=great difficulty) |  |  |  |  |  |
| some difficulty | .97 | .97 | .94 | .94 | .94 |
| fairly easily | **.84*** | **.84**** | **.80**** | **.80**** | **.80**** |
| easily | **.77**** | **.77***** | **.76***** | **.76***** | **.76***** |
| Living with partner | **.33*** | **.33***** | **.39***** | **.39***** | **.39***** |
| Mobility limited | **1.26***** | **1.26***** | **1.33***** | **1.33***** | **1.33***** |
| Retired | .93 | .93 | .91 | .90 | .91 |
| Test positive Covid | 1.01 | 1.01 | 1.02 | 1.02 | 1.02 |
| Country (Ref=Greece) |  |  |  |  |  |
| Israel | **1.48*** | 1.46* | **.43***** | **.43***** | .45*** |
| **Occidental** |  |  |  |  |  |
| Austria | **1.51**** | **1.52**** | .26*** | **.26***** | **.27***** |
| Belgium | **1.81***** | **1.81***** | **.24***** | **.24***** | **.25***** |
| France | .93 | .93 | **.57***** | **.56***** | **.58***** |
| Germany | **1.70***** | **1.71***** | **.42***** | **.42***** | **.43***** |
| Luxembourg | **1.89***** | **1.88***** | **.52***** | **.52***** | .52*** |
| Netherlands | **1.53**** | **1.53**** | **.263**** | **.25***** | **.27***** |
| Switzerland | .95 | .96 | **1.62***** | **1.61***** | **1.62***** |
| **North** | **1.82***** | **1.82***** | 1.17 | **1.14***** | 1.16 |
| Denmark | **4.35***** | **4.38***** | **.20***** | **.20***** | **.21***** |
| Estonia | **1.27*** | **1.28*** | **.39***** | **.39***** | .39*** |
| Finland | 1.17 | **1.17***** | .76 | .74 | .76 |
| Latvia | **2.25***** | **2.28***** | **.36***** | **.36***** | .37*** |
| Lithuania | **1.44**** | **1.46**** | .98 | .98 | .99 |
| Sweden | 1.33 | 1.32 | **.41***** | **.41***** | **.42***** |
| **South** | **1.69***** | **1.70***** | **.54***** | **.53***** | .54*** |
| Croatia | 2.43 | **2.46***** | **.45***** | **.45***** | .46*** |
| Italy | **1.82***** | **1.82***** | 1.17 | **1.14***** | 1.16 |
| Malta | **2.23***** | **2.22***** | .85 | .85 | .87 |
| Slovenia | 1.17 | 1.18 | **.66*** | **.65*** | .66* |
| Spain | **3.23***** | **3.20***** | **.47***** | **.46***** | **.47***** |
| Cyprus | **2.24***** | **2.26***** | .93 | .91 | .96 |
| **East** | **1.77***** | **1.77***** | **.50***** | **.51***** | .51*** |
| Bulgaria | **1.77***** | **1.77***** | **.50***** | **.51***** | .51*** |
| Czech Republic | **1.64***** | **1.63***** | **.62**** | **.60**** | .62** |
| Hungary | 1.32 | 1.31 | **.51***** | **.50***** | .52*** |
| Poland | **1.69***** | **1.70***** | **.54***** | **.53***** | .54*** |
| Romania | **1.79***** | **1.80***** | **.50**** | **.49**** | **.51**** |
| Slovakia | **3.31***** | **3.34***** | **.54***** | .**54***** | **.54***** |
| N | 17915 | 17929 | 17841 | 17850 | 17847 |

*Note*: Loneliness baseline was added as additional control variable for robustness check. * p < 0.05; ** p < 0.01; *** p < 0.001.

Supplemental Table 3. Odds ratio of changes in volunteering over time

| Ref. No volunteer at any time | **Lonely T1** | **Lonely T2** |
| --- | --- | --- |
| Volunteer T0 | **.86**** | .93 |
| Volunteer T1 | .90 | 1.11 |
| Volunteer T0 T1 | .83 | .88 |
| Volunteer T2 |  | **.75**** |
| Volunteer T0 T2 |  | **.61***** |
| Volunteer T1 T2 |  | .97 |
| Volunteer at all times |  | **.69*** |
| Age | **1.02***** | **1.02***** |
| Women | **1.42***** | **1.51***** |
| Education | .99 | .99 |
| Ends meet (Ref=great difficulty) |  |  |
| some difficulty | **.86*** | **.85*** |
| fairly easily | **.71***** | **.69***** |
| easily | **.62***** | **.62***** |
| Living with partner | **.27***** | **.32***** |
| Mobility limited | **1.42***** | **1.48***** |
| Retired | **.99***** | **.96***** |
| Test positive Covid | .99 | **.98***** |
| Country (Ref=Greece) |  |  |
| Israel | **.43***** | **.61**** |
| **Occidental** |  |  |
| Austria | **.22***** | **.21***** |
| Belgium | **.47***** | **.40***** |
| France | **.51***** | **.55***** |
| Germany | **.37***** | **.36***** |
| Luxembourg | .46*** | .44*** |
| Netherlands | **.36***** | **.26***** |
| Switzerland | **.27***** | **.24***** |
| **North** |  |  |
| Denmark | **.21***** | **.17***** |
| Estonia | **.31***** | **.37***** |
| Finland | **.33***** | **.34***** |
| Latvia | **.59***** | **.73*** |
| Lithuania | **.36***** | **.43***** |
| Sweden | **.44***** | **.37***** |
| **South** |  |  |
| Croatia | **.68**** | .92 |
| Italy | **1.01** | 1.17 |
| Malta | **.59**** | **.45***** |
| Slovenia | **.28***** | **.31***** |
| Spain | **.38***** | **.39***** |
| Cyprus | **.64*** | .72 |
| **East** |  |  |
| Bulgaria | **.49***** | .87 |
| Czech Republic | **.40***** | **.44***** |
| Hungary | **.32***** | **.54***** |
| Poland | **.43***** | **.45***** |
| Romania | **.50***** | **.51***** |
| Slovakia | **.22***** | **.21***** |
| N | 17954 | 17908 |

*Note*: T1 (time 1, SHARE Covid Survey 1), T2 (time 2, SHARE Covid Survey 2) * p < 0.05; ** p < 0.01; *** p < 0.001.

Supplemental Table 4. Robustness check controlling for baseline loneliness.

| Ref. No volunteer at any time | **Lonely T1** | **Lonely T2** |
| --- | --- | --- |
| Loneliness baseline | **3.55***** | **3.30***** |
| Ref = No volunteer at any time |  |  |
| Volunteer T0 | .84 | .98 |
| Volunteer T1 | .91 | 1.10 |
| Volunteer T0 T1 | .88 | .86 |
| Volunteer T2 |  | **.73**** |
| Volunteer T0 T2 |  | **.63***** |
| Volunteer T1 T2 |  | .96 |
| Volunteer at all times |  | **.70*** |
| Age | **1.02***** | **1.02***** |
| Women | **1.46***** | **1.56***** |
| Education | 1.01 | 1.01 |
| Ends meet (Ref=great difficulty) |  |  |
| some difficulty | .97 | .96 |
| fairly easily | **.84*** | **.80**** |
| easily | **.77***** | **.77***** |
| Living with partner | **.33***** | **.33***** |
| Mobility limited | **1.26***** | **1.34***** |
| Retired | **.93***** | **.92***** |
| Test positive Covid | 1.02*** | 1.00 |
| Israel | **.45***** | **.61**** |
| **Occidental** |  |  |
| Austria | **.30***** | **.27***** |
| Belgium | **.55***** | **.45***** |
| France | **.55***** | **.59***** |
| Germany | **.46***** | **.43***** |
| Luxembourg | **.57**** | **.53***** |
| Netherlands | **.40***** | **.28***** |
| Switzerland | **.29***** | **.25***** |
| **North** |  |  |
| Denmark | **.28***** | **.21***** |
| Estonia | **.38***** | **.45***** |
| Finland | **.36***** | **.37***** |
| Latvia | **.68*** | .84 |
| Lithuania | **.44***** | **.51***** |
| Sweden | **.51***** | **.42***** |
| Greece | **1.31*** | **1.64***** |
| **South** |  |  |
| Croatia | **.73*** | .99 |
| Italy | .98 | 1.14 |
| Malta | .67 | **.50*** |
| Slovenia | **.35***** | **.40***** |
| Spain | **.46***** | **.47***** |
| Cyprus | .68 | .75 |
| **East** |  |  |
| Bulgaria | **.54***** | .95 |
| Czech Republic | **.50***** | **.54***** |
| Hungary | **.40***** | .67* |
| Poland | **.51***** | **.52***** |
| Romania | **.54***** | **.54***** |
| Slovakia | **.30***** | **.27***** |
| Bulgaria | **.54***** | .95 |
| N | 17913 | 17866 |

*Note*: This is an additional control variable added as a robustness check. * p < 0.05; ** p < 0.01; *** p < 0.001.
